# Phages indirectly maintain tomato plant pathogen defense through regulation of the commensal microbiome

**DOI:** 10.1093/ismeco/ycaf065

**Published:** 2025-04-18

**Authors:** Reena Debray, Asa Conover, Britt Koskella

**Affiliations:** Department of Primate Behavior and Evolution, Max Planck Institute for Evolutionary Anthropology, 04103 Leipzig, Germany; Department of Integrative Biology, University of California, Berkeley, 94720 Berkeley, CA, United States; Department of Integrative Biology, University of California, Berkeley, 94720 Berkeley, CA, United States; Department of Integrative Biology, University of California, Berkeley, 94720 Berkeley, CA, United States

**Keywords:** phage–bacteria interactions, microbiome–host interactions, phytopathogen, kill the winner, phage depletion

## Abstract

As parasites of bacteria, phages can regulate microbiome diversity and composition and may therefore affect susceptibility to pathogens and disease. Many infectious diseases are associated with altered bacteriophage communities, but observational studies alone do not allow us to determine when altered phage community composition is a contributor to disease risk, a response to infection, or simply an indicator of dysbiosis. To address this question directly, we used size-selective filtration to deplete plant-associated microbial communities of phages, then challenged plants with the bacterial pathogen *Pseudomonas syringae*. Plants with phage-depleted microbiomes were more susceptible to infection, an effect that could not be explained by direct effects of the phage communities on either *P. syringae* or the plant host. Moreover, the presence of phages was most impactful when the phage communities were isolated from neighboring field locations rather than from the same host plant as the bacteria, possibly suggesting that moderate rates of lysis maintain a community structure that is most resistant to pathogen invasion. Overall, our results support the idea that phage communities contribute to plant defenses by modulating the microbiome.

## Introduction

Viruses that infect bacteria, or phages, shape microbial ecosystems in significant ways, such as transferring genetic material among bacterial strains, altering growth rates and competition dynamics of their hosts, and releasing nutrients sequestered in bacterial cells [1–4]. Some phages infect and kill bacteria that are pathogenic to plants or animals, and these phages have long been recognized as an opportunity for managing human and agricultural diseases due to their specificity and their potential to self-replicate and co-evolve with resistant bacteria [5–7]. In comparison, phages that do not directly target bacterial pathogens have received substantially less attention. However, many diseases of animals and plants are characterized by altered phage communities [8–11], suggesting that they may also modulate disease risk. Just as it has historically been challenging to link bacterial community composition to disease phenotypes, it is difficult to tell from observational data alone whether an altered phage community is a cause, a consequence, or simply an indicator of a disease state [12–14]. Experiments that directly manipulate phage presence and/or composition are therefore needed to identify whether and how phages shape disease susceptibility.

**Figure 1:**
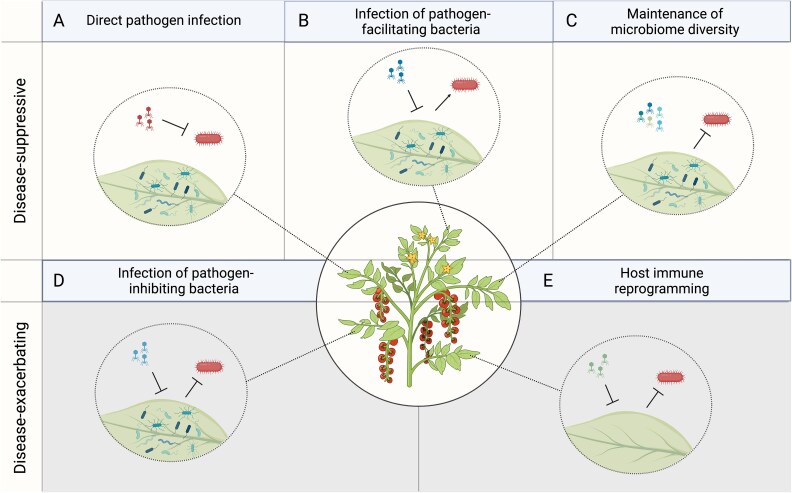
Possible mechanisms of phage modulation of pathogen infection. Phages may suppress disease by (A) directly reducing pathogen population sizes [5–7, 66–68], (B) reducing the population sizes of microbiome members that facilitate pathogen infection, or (C) restricting dominant bacteria and in turn facilitating other protective bacterial species that would have otherwise gone extinct. Phages may facilitate disease by (D) reducing the population sizes of protective microbiome members [10], or (E) reprogramming host immunity to trigger an antiviral response and suppress the antibacterial response (currently documented only in mammalian hosts, [18]). Diagrams were generated using BioRender.

A relationship between phage activity and disease could arise through several pathways that are not mutually exclusive (Fig. 1). One possibility is that direct recognition of phages by the immune system of the plant or animal host alters immune defenses against pathogens. Phages share some molecular features with mammalian viruses [15] and have been shown to promote antiviral cytokine production in humans and mice [16, 17]. In one case, a filamentous phage of *Pseudomonas aeruginosa* triggered an antiviral pathway that suppressed phagocytosis of bacterial cells, exacerbating the *P. aeruginosa* infection [18]. Another possibility is that phage-mediated lysis of bacteria triggers an immune response to the contents of the bacterial cells. For example, lysis can release lipopolysaccharide (LPS), a component of the bacterial membrane that is recognized by plant and animal immune systems [19–22].

Phages may also influence disease outcomes by altering the composition of the resident microbiome. The ability of microbial pathogens to establish in hosts and cause disease symptoms often depends on interactions with other microbiome members. For instance, resident bacteria with similar ecological niches to pathogens can limit pathogen colonization through competition [23, 24]. Other microbiome members can activate or suppress plant defenses [25–27] or produce compounds that directly inhibit pathogens [28, 29]. The resident phage community may therefore influence disease susceptibility by altering the abundances of pathogen-facilitating or pathogen-inhibiting bacteria. For example, variation in the severity of bacterial wilt disease in the tomato rhizosphere was attributed to the presence of pathogen-inhibiting bacteria and their associated phages. Tomato plants inoculated with inhibitory bacteria were more resistant to bacterial wilt, but this protective effect vanished in the presence of inhibitor-associated phages [10].

A particularly important feature of phages for disease is their strain- or species-specific killing ability. Ecological models predict that any surge in the abundance of a bacterial species is followed by a surge in its specialized phages, which then reduce the population size of the formerly abundant bacteria [30–34]. By restricting dominant bacteria, phages may open niches for other, possibly protective, bacteria that would have otherwise remained rare or gone extinct. In support of this hypothesis, bacteria in several habitats form more species-rich and synchronous communities when combined with a diverse set of phages than in the absence of phages [35–37].

In this study, we asked whether phages can modulate plant disease susceptibility, and if so, whether they do so by directly infecting pathogenic bacteria or by reshaping the commensal microbiome. We isolated microbial communities from the leaves (phyllosphere) of field-grown tomato plants and depleted them of phages using size-selective filtration. We transplanted cellular microbes either with or without their (or other) phage fractions onto tomato leaves in a series of growth chamber experiments. This approach is analogous to predator exclusion experiments that have been conducted in macro-scale ecosystems to determine how top-down processes shape community structure [38, 39]. We then challenged the plants with *Pseudomonas syringae,* a bacterial pathogen with pathovars infecting nearly 200 plant species, including a number of economically important crops such as wheat, barley, soybean, kiwifruit, and tomato [40, 41]. Previous research shows that applying a supplemental microbiome to tomato leaves can limit subsequent *P. syringae* growth and symptom progression [29, 42, 43], indicating a role for the phyllosphere microbiome in disease susceptibility. We report here that this protective effect is attenuated in the absence of the phage communities. We further show that the contribution of phages to pathogen reduction was not explained by direct effects of the phage communities on *P. syringae* alone. Rather, the effect of the phage fractions was contingent on the presence and identity of their microbial hosts.

## Materials and methods

### Microbiome sampling from field-grown plants

To assess the role of phages in microbiome-mediated reduction of pathogen colonization, leaves were sampled from six different rows of six-month-old tomato plants at the Student Organic Farm at the University of California, Davis (38°32′20.04″ N, 121°44′57.36″ W) in September 2018. Leaves were collected from the full range of the height of the plants and placed in a loosely filled bag (35–50 grams of plant material). Bacteria and phage communities were isolated from leaves within one week of field sampling.

To validate and extend the results of the first field season, leaves were sampled from 10 six-month-old tomato plants at the Student Organic Farm at the University of California, Davis (38°32′31.2″ N, 121°45′46.8″ W) in August 2021. These plants were used to generate inocula for a second phage depletion experiment with the same treatments and conditions as the 2018 experiment, but with a different strain of the pathogen *P. syringae*. In the same month, 6 tomato plants were sampled from the Student Organic Farm and 6 tomato plants and 6 American black nightshade plants (*Solanum americanum*) were sampled from the Vegetable Crops Field (38° 32′ 20.5″ N, 121° 46′ 54.7″ W). These plants were used to generate inocula for an experiment that paired phage communities with either their own bacterial communities or bacterial communities from other sources.

### Isolation of microbial and phage communities

A buffer solution of 10 mM MgCl_2_ was added to each bag to cover the leaves, and the Ziploc bags were submerged in a Brandon M5800 sonicating water bath for 10 min to gently dislodge microbial cells from the leaf surface. The resulting leaf wash was passed through a 20 μm membrane to remove plant tissue, then vacuum filtered through a 0.2 μm membrane to separate cellular microbes from viral particles.

To release cellular microbes such as bacteria, archaea, and fungi (hereafter referred to as “microbial communities” or “phage-depleted microbial communities”), the 0.2 μm filter was transferred to a sterile tube and sonicated in 10 mM MgCl_2_. Microbial cells were pelleted at 3500 x *g* for 10 min, resuspended in 50% glycerol, and stored at −80°C. The filtrate from the 0.2 μm filter, containing viruses and small molecules, was transferred to an Amicon Ultra-15 filter unit with a 100 kDa molecular weight cutoff. Amicon filters were centrifuged at 4000 x *g* for 25 min to isolate and concentrate viruses. The resulting phage fraction was stored at 4°C.

This method of phage isolation and concentration was originally developed to isolate viruses in seawater [44, 45] and has been previously adapted to the tomato phyllosphere [35]. It separates the majority of lytic phages from their hosts, but lysogenic phages or lytic phages actively infecting a bacterial cell at the moment of filtration may remain in the bacterial fraction, thus resulting in a “phage-depleted” rather than an entirely phage-free microbial community. However, in a previous application of this method to tomato leaf microbiomes, no infectious phage particles were ever detected within phage-depleted microbial communities, and total phage deoxyribonucleic acid (DNA) was reduced to 0.1%–1.3% of its original concentration on tomato leaves [35].

### Phage depletion experiments

To measure the effect of phage depletion on microbiome-mediated resistance to pathogen colonization, microbial inocula were applied to plant leaves, either with or without their respective phage communities. Early Girl tomato seeds (Eden Brothers) were surface sterilized in 70% ethanol for 1 min, then in 10 ml of 6% bleach and 10 ml of 0.2% Tween 20 for 20 min. Seeds were placed in a petri dish containing 0.8% water agar, then covered and incubated at 21°C in the dark. After germination, plates were maintained in a growth chamber at 24°C and 70% humidity with a 15 h day:9 h night light cycle. Nine days after planting, seedlings were transferred to pots containing autoclaved potting medium (Profile Porous Ceramic Greens Grade soil amendment, Sierra Pacific Turf Supply). Pots were spatially randomized with respect to treatment for the duration of the experiment.

Three weeks after planting, leaves were sprayed with microbial communities from the field. Microbiome and phage inocula were each standardized to a fixed mass of plant material from the field (6 grams) so that phage-host ratios in the inocula were similar to those in their natural environment. Inocula were prepared in 4 ml of 10 mM MgCl_2_, with 0.04 μl of the surfactant Silwet L-77 added to facilitate microbial adhesion to the leaf surface. Leaves were sprayed from all angles using a 15 ml conical tube fitted with a spray cap.

Four weeks after planting (one week after microbiome inoculation), leaves were challenged with *P. syringae* pv tomato. This timing was selected based on previous observations that within one week after spray-inoculation, bacterial communities reach comparable population densities to the native microbiomes of natural plant populations [35]. Two different strains of *P. syringae* were used in separate experiments: DC3000, a model plant pathogen that infects *Arabidopsis thaliana* as well as tomato plants, and PT23, a closely related pathovar that is more specialized to tomato plants [46]. In both cases, an overnight culture of *P. syringae* was pelleted and diluted to an optical density (OD_600_) of 0.0002. The resulting microbial suspension was infiltrated into the abaxial side of the leaves (three per plant) using a blunt-end syringe. At 24 h post-infection, three hole punches (6-mm diameter) were taken from each leaf. Leaf discs were homogenized in 1 ml 10 mM MgCl_2_ in a FastPrep-24 5G sample disruption instrument at 4.0 m/s for 40 s and stored at −20°C for molecular analysis.

Healthy leaves from each plant (i.e. not challenged with *P. syringae*) were collected, suspended in 10 mM MgCl_2,_ and sonicated, pelleted, and frozen as described above. These leaf samples served several purposes. First, they allowed us to characterize the effects of phage depletion on the commensal microbial communities. Second, they provided a baseline quantification of other pathogenic or non-pathogenic *P. syringae* strains that were present in the plant microbiome prior to the pathogen challenge (note that a true baseline sample of the same leaf before and after infection is not possible in this system, as profiling the microbiome requires destructive sampling of the leaf). Droplet digital polymerase chain reaction (PCR) detected 0–15 copies of the *Pseudomonas* sequence per 6-mm leaf disc in comparison to ~10 000 per leaf disc in infected leaves, making it extremely unlikely that any differences among treatments were driven by other *P. syringae* strains (Table S1).

### Lipopolysaccharide quantification

The Pierce Chromogenic Endotoxin Quant Kit (ThermoFisher Scientific Cat. #A39553) was used to measure LPS concentrations in the phage filtrates. Amebocyte lysate that binds to LPS was added to samples and incubated at 37°C for 30 min. A chromogenic substrate that reacts with the amebocyte proenzyme was added and incubated at 37°C for 6 min. Optical density values were recorded at 405 nm.

To assess whether LPS accumulation was responsible for the observed effect of phage on disease outcomes *in planta*, the leaves of three-week-old tomato plants were sprayed with either phage communities or varying concentrations of pure LPS from *Escherichia coli.* Leaves were challenged one week after spraying with *P. syringae* and harvested as described above.

### Reciprocal transplant experiment

Tomato plants were grown and maintained in the growth chamber as described above. The following inocula were applied to three-week-old plants: (i) microbiome with sympatric phage communities (isolated from the same plant), (ii) microbiome with allopatric phage communities (isolated from a neighboring tomato plant), (iii) microbiome with allopatric phage communities (isolated from a different plant species, American black nightshade, in the same field), (iv) microbiome with allopatric phage communities (isolated from tomato plants in a different field, approximately 2 km away), and (v) microbiomes depleted of phages. The same six microbiome communities were used in all five treatments, with only the phage communities changing. One week after microbiome inoculation, leaves were challenged with *P. syringae* and harvested as described above.

### Test for direct phage infectivity

To test whether phage communities contained any phages capable of directly infecting *P. syringae*, co-cultures of *P. syringae* and 100 μl of each phage fraction were incubated in King’s B Broth for 24 h at 28°C. The resulting overnight culture was passed through a 0.2 μm filter to isolate any phages that might have amplified in the presence of *P. syringae*. Next, 200 μl of *P. syringae* overnight culture was mixed with 2 ml of King’s B Broth supplemented with 0.6% agar. The soft agar mixture was spread evenly onto petri dishes and allowed to dry, then 30 μl of filtrate from the overnight culture was pipetted on top. Plates were incubated at 28°C and monitored daily for signs of bacterial lysis.

### Quantification and analysis of phytopathogen population sizes

*P. syringae* population sizes on leaves were measured using the Bio-Rad QX200™ Droplet Digital PCR system (see Tables S2–S3 for primer sequences and cycling conditions). This measure is highly correlated with estimates of *P. syringae* population sizes based on colony counts on agar plates, and unlike plating, can be performed after samples are preserved in the freezer and/or repeated if needed from the same samples [47]. Samples were randomized on the plates, with a no-template control in the last well of each column. Droplet thresholds were set by column based on the fluorescence values in the range of the negative control.

The ability of phage depletion to limit *P. syringae* colonization was assessed using linear regression. For each experiment, the dependent variable was the copies of *P. syringae* detected per standardized disc of infected leaf tissue, which was approximately normally distributed and had similar variance among treatments. The independent variable was the treatment, with the positive control (plants sprayed with a sterile buffer solution in place of a supplemental microbiome prior to *P. syringae* challenge) as the reference level. In the experiment testing the effect of pure LPS on *P. syringae* colonization, the dependent variable was again the number of copies of *P. syringae* per leaf disc and the independent variable was the concentration of LPS (endotoxin units per milliliter). In the reciprocal transplant experiment, the effect of different phage communities on *P. syringae* was assessed using a paired *t*-test, with pairs of samples linked if they were treated with the same microbial community but different phage communities. This model structure controlled for the fact that microbial communities from different field sources had different intrinsic resistance to *P. syringae* colonization. It tested whether the identity of the phage community (sympatric or allopatric) affected the intrinsic resistance levels of the various microbial communities in the same direction.

### Deoxyribonucleic acid extraction and sequencing

DNA was extracted from leaf wash filtrate from the field plants (i.e. the inocula used for the reciprocal transplant experiments), as well as from the following treatments of the reciprocal transplant experiment: microbiomes with sympatric phages present, and microbiomes transplanted with phages isolated from a neighboring tomato plant in the field, and microbiomes depleted of phages. Extractions were performed using the DNeasy PowerSoil kit. Libraries were prepared by amplifying the V4 region of the 16S ribosomal ribonucleic acid (rRNA) gene. Libraries were amplified, cleaned, and sequenced alongside DNA extraction controls and PCR controls on the Illumina MiSeq platform at Microbiome Insights (Vancouver, BC, CAN).

Reads were analyzed using the recommended DADA2 workflow [48] to infer amplicon sequencing variants. Forward reads were truncated at position 240 and reverse reads were truncated at position 140. Reads were filtered to allow 2 or fewer expected errors on the forward reads and 5 or fewer expected errors on the reverse reads. Taxonomy was assigned using the SILVA database [49] and all sequences mapping to plant chloroplast or mitochondria were removed. To compare the effect of phage communities on microbiome composition, a paired *t*-test was again used to control for the variation among microbial communities from different field sources to phage treatment. The dependent variable was the Bray–Curtis distance between the final microbiome composition at harvest when it was transplanted with phages versus without.

### Quantification of total bacterial abundance

As the amplicon sequencing data revealed high concentrations of chloroplast DNA in our leaf wash samples, we reasoned that accurately quantifying total bacterial abundance through droplet digital PCR would require an additional effort to exclude host DNA. We selected primers to target a region of the 16S rRNA gene that is conserved among bacteria, but has several mismatches with plant chloroplast and mitochondrial 16S rRNA sequences [50] (see Tables S4–S5 for primer sequences and cycling conditions). Samples were randomized on the plates, with a no-template control in the last well of each column. Droplet thresholds were set by column based on the fluorescence values in the range of the negative control. We used known mixtures of bacterial culture and pure extracted chloroplasts to verify that this primer set reduced chloroplast amplification by 90–95% compared to standard 16S V4 primers, while still amplifying the majority of bacteria detected by the standard primers (Fig. S1).

## Results

### Phage depletion reduces microbiome-mediated protection

To assess the role that the phage component of the microbiome plays in colonization of a bacterial pathogen, we sampled phyllosphere microbial communities from six different rows of an organic tomato farm. We isolated microbial communities from the leaves using an ultrasonic bath, then separated cellular microbes from viruses and small molecules using a series of size-selective filters. We then transplanted microbial communities, with or without their respective phages, onto juvenile tomato plants in the lab. After one week, we challenged plants with the bacterial pathogen *P. syringae.* We measured *P. syringae* population sizes (a correlate of disease epidemiology and plant susceptibility to frost damage [51]) after an additional 24 h using droplet digital PCR [52].

**Figure 2:**
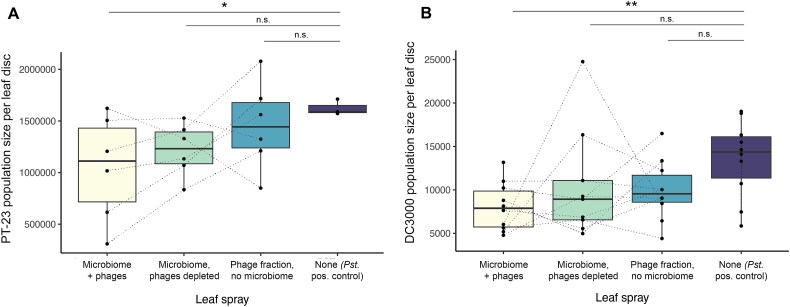
Supplemental microbiome and phages reduce *Pseudomonas syringae* colonization of tomato leaves in two independent harvest seasons. (A) Population sizes of *P. syringae* pv. *Tomato* strain PT-23 on plants sprayed with microbial and phage communities collected from an organic farm in 2018. (B) Population sizes of *P. syringae* pv. *Tomato* strain DC3000 on plants sprayed with microbial and/or phage communities collected from an organic farm in 2021. Box-plots lines indicate the lower quartile, median, and upper quartile, and whiskers indicate 1.5 times the interquartile range. Asterisks represent statistical significance: 0.001 < *P* < .01 (**); 0.01 < *P* < .05 (*). Dashed lines connect points that originated from the same source microbiome in the field.

The microbial inocula, isolated from six different locations around the farm, varied in their relative conferred resistance to pathogen colonization. Plants sprayed with the same microbial communities, whether in the presence or absence of phages, had correlated pathogen loads (Pearson’s correlation, df = 4, *P* = .013, Fig. S2). Because we chose to use biological replicates to capture natural variation in microbiome composition, we did not determine what explains this variation, and indeed this was not the goal of the current work. Instead, we sought to ask whether these variable microbial communities differed, on average, in their disease protective effects when the phages were depleted. Across biological replicates, *P. syringae* consistently reached lower population sizes when phages were present than when they were depleted (paired *t-*test, t = 2.82, df = 5, *P* = .037). *P. syringae* population sizes were approximately 35% lower in the “microbiome + phages” treatment compared to control plants that were sprayed with only a sterile buffer prior to infection (t = 2.11, df = 17, *P* = .049, Fig. 2A). Neither phage-depleted microbial communities, nor phage fractions without their microbial hosts, were significantly protective against *P. syringae*, suggesting that the effect depended on phage-host interactions.

To generalize the results of the first experiment, we collected tomato leaves in a subsequent harvest season and repeated the phage depletion process. We sprayed tomato plants with microbial communities as before, but this time we challenged the leaves with a generalist strain of the same species, *P. syringae* pv. *tomato* strain DC3000. Compared to the more specialized strain PT-23, DC3000 reached lower population sizes on tomato plants. Control plants that were treated with only DC3000 harbored 10^2^–10^4^ copies of the *Pseudomonas*-specific marker gene sequence per 6-mm leaf disc, compared to 10^6^ copies per disc in control plants infected with PT-23. Though treatment differences were smaller in this trial, *P. syringae* populations were again significantly reduced in plants treated with microbial and phage communities together, compared to either control plants (t = 2.81, df = 35, *P* = .008), or plants treated with phage-depleted microbial communities (t = 2.17, *P* = .048) (Fig. 2B).

### Direct phage infection of pathogen does not explain the pathogen reduction effect

We first considered the possibility that phage communities aided in limiting pathogen population growth because they contained, by chance, phages capable of directly infecting *P. syringae*. This seemed unlikely given that phage communities were not significantly protective on their own. However, if free phages decayed rapidly in the week between microbiome treatment and pathogen challenge, it is possible that the presence of their microbial hosts helped to maintain sufficient phage populations to subsequently infect and limit *P. syringae*.

**Figure 3:**
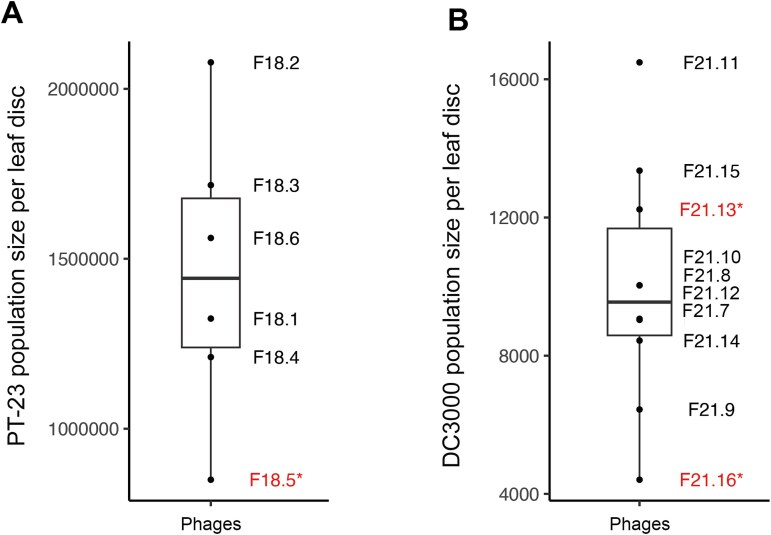
Identifying *P syringae*-targeting phage activity in field-collected phage communities. (A) Population sizes of *P. syringae* pv. *Tomato* strain PT-23 on plants treated with phage communities collected from an agricultural field plot in 2018. (B) Population sizes of *P. syringae* pv. *Tomato* strain DC3000 on plants treated with phage communities collected from an agricultural field plot in 2021. Phage communities in red with an asterisk indicate plaque-forming activity on agar plates of the respective strain of *P. syringae.* Box-plots lines indicate the lower quartile, median, and upper quartile, and whiskers indicate 1.5 times the interquartile range.

To test this possibility, we co-cultured each phage community with each strain of *P. syringae* to amplify any infective phages if they were present, then plated the co-culture filtrate onto bacterial lawns of *P. syringae*. Only one of the six phage communities in the first harvest season, F18.5, produced plaques on the *P. syringae* plates. This phage community was associated with unusually low *P. syringae* growth in the plant experiment as well, suggesting that a *P. syringae*-targeting phage in this community may have directly reduced *P. syringae* colonization *in planta* (Fig. 3A). Of note, the statistical effect of the “microbiome + phages” treatment did not change when the data were reanalyzed to exclude site F18.5 (t = 2.73, df = 14, *P* = .016), indicating that the protective effect was not solely driven by direct infection by a single phage.

In the second harvest season, two out of ten phage communities contained phages capable of infecting *P. syringae*, including the community associated with the lowest *P. syringae* colonization in the plant experiment (Fig. 3B). Again, the effect of the “microbiome + phages” inocula remained significant when these field sources were excluded from analysis (t = 2.31, df = 35, *P* = .029). These results indicate that although direct phage infection of *P. syringae* may have occurred occasionally in our experiments, it was not widespread and could not fully account for the effect of phages on pathogen reduction.

### Lipopolysaccharide accumulation in filtrate does not explain the observed reduction in pathogen colonization

We next considered the possibility that the phage depletion process may have accumulated other particles in addition to viruses. In particular, ultrafiltration has the known side effect of concentrating LPS, a component of the outer membranes of Gram-negative bacteria [53]. If LPS molecules in the phage filtrates triggered a plant immune response, such an effect could be mistakenly attributed to a protective effect of phages. We note that this explanation seemed unlikely, as it expects the phage communities to limit *P. syringae* growth even in the absence of their respective microbial communities, which was not the case in our plant experiments. Nevertheless, we sought to exclude this possibility by quantifying the LPS concentration in the phage filtrates.

LPS concentrations in our phage filtrates ranged from 1–3.5 endotoxin units per milliliter (EU/ml), which fall on the low end of the typical range of reported LPS levels of phage preparations in other studies [53–55]. When we sprayed tomato plants with pure bacterial LPS in this range of concentrations, we found no relationship between the concentration of pure LPS applied to the plant leaves and the subsequent colonization of *P. syringae* (t = 0.627, df = 3, *P* = .575, Fig. S3A). Furthermore, plants that were treated with LPS (at any concentration) prior to the pathogen challenge had similar infection outcomes to plants that were not (t = 0.262, df = 6, *P* = .802). Finally, there was no relationship between the LPS concentrations of the phage filtrates and their ability to limit *P. syringae* colonization (t = −0.48, df = 4, *P* = .656, Fig. S3B).

### Phage contributions to plant pathogen defense depend on microbiome identity

**Figure 4:**
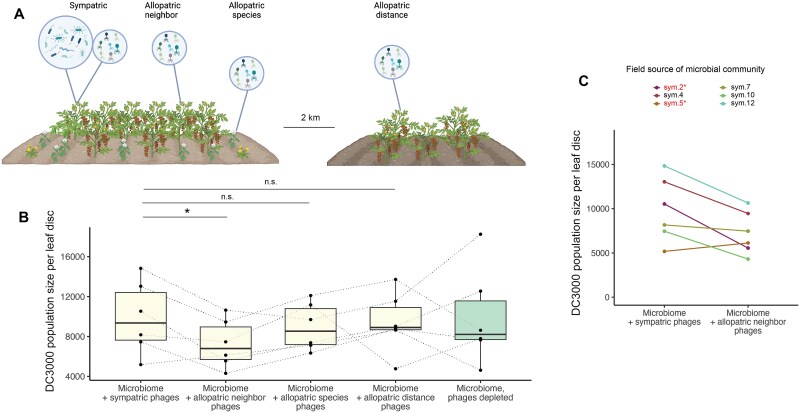
Phages from a neighboring plant more effectively reduce *Pseudomonas syringae* colonization of tomato leaves than phages from the same plant as the supplemental microbiome. (A) Diagram showing how phage communities were collected to generate the “allopatric neighbor”, “allopatric species”, and “allopatric distance” treatments. (B) Population sizes of *P. syringae* pv. *Tomato* strain DC3000 on plants treated with microbial and/or phage communities collected from an agricultural field plot in 2021. Asterisks represent statistical significance: 0.001 < *P* < .01 (**); 0.01 < *P* < .05 (*). Dashed lines connect points that originated from the same source microbiome in the field. Box-plots lines indicate the lower quartile, median, and upper quartile, and whiskers indicate 1.5 times the interquartile range. (C) Population sizes of *P. syringae* pv. *tomato* strain DC3000 on plants treated with microbial and “sympatric” or “allopatric neighbor” phage communities, colored by the identity of the microbial community. Red text with an asterisk indicates plaque-forming activity on agar plates of the respective strain of *P. syringae.* Lines connect points that originated from the same source microbiome in the field. Diagrams were generated using BioRender.

We next explored whether the ability of phages to limit *P. syringae* colonization depended on interactions with microbial hosts; for example, by altering competitive dynamics or species diversity within the bacterial microbiome. We sprayed microbial and phage communities from tomato plants in the field onto juvenile tomato plants in the lab. A set of six focal microbial communities were paired in turn with (i) phage fractions from the same tomato plant in the field, as was the case for all preceding experiments (“sympatric”), (ii) phage fractions from a different tomato plant in the same field (“allopatric neighbor”), (iii) phage fractions from an American black nightshade plant in the same field (“allopatric species”), (iv) phage fractions from a tomato plant grown in a different field (“allopatric distance”) (Fig. 4A).

Microbiomes paired with phage communities from neighboring tomato plants were consistently more protective against *P. syringae* DC3000 than microbiomes paired with phage communities from their own plant (paired *t*-test, t = 2.82, *P* = .037, Fig. 4B). The sole exception to this pattern, phage source “sym5”, contained a *P. syringae*-targeting phage, suggesting that it deviated from the pattern because it directly reduced *P. syringae* colonization whether it was paired with its own microbial community or another one (Fig. 4C). The difference between phages from neighboring plants and phages from the same plant remained significant when excluding “sym5” and the other field source with a *P. syringae* phage (paired *t*-test, t = 3.80, *P* = .032). The two more distant forms of allopatry, phage communities from a different plant species or from a different field, were no different than sympatric phage communities with respect to protectiveness (*P* > 0.05).

**Figure 5:**
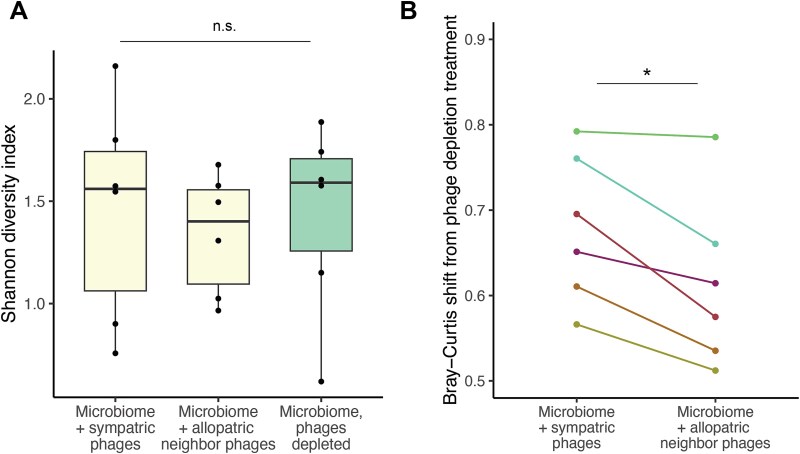
Composition of microbial communities after transplant with or without phages. (A) Shannon diversity of microbial communities in each treatment from the sympatric and allopatric transplant experiment. (B) Bray–Curtis dissimilarity of microbial communities in each “microbiome + phages” treatment compared to “microbiome, phages depleted” plants. Higher values indicate more community turnover. Asterisks represent statistical significance: 0.001 < *P* < .01 (**); 0.01 < *P* < .05 (*). Lines connect points that originated from the same source microbiome in the field.

To understand why phages from neighboring plants reduced pathogen colonization more than phages from the same plant as the bacteria, we sequenced the 16S V4 amplicon of the microbial communities that had been transplanted with either sympatric or “allopatric neighbor” phage communities. These treatments did not differ from each other or from phage-depleted microbiomes in their alpha diversity (Fig. 5A). However, compared to phage-depleted microbiomes, transplanting with sympatric phages shifted the final composition of each bacterial community more than transplanting with allopatric phages (Fig. 5B, paired t = 3.85, df = 5, *P* = .012). This was in line with phages being, on average, locally adapted to their bacterial hosts and suggests that more bacterial infections took place in the sympatric pairing. Using droplet digital PCR to measure the total abundance of the bacterial community on the leaves, we found that sympatric phages significantly reduced bacterial abundance compared to phage-depleted microbiomes (Fig. S4, t = −4.44, *P* = .001) and marginally reduced bacterial abundance compared to allopatric phages (t = −1.75, *P* = .089).

## Discussion

Historically, the majority of clinical microbiology research and interventions focused on bacterial pathogens until the relatively recent expansion of microbial ecology revealed that commensal microbiome regulation plays an important role in immune health as well [56, 57]. In a similar vein, though phages have primarily attracted interest in medical microbiology for their ability to infect bacterial pathogens, our work and other recent studies highlight phages of commensal bacteria as an important component of microbiome function [10, 58]. We found that microbiome and phage communities, when applied to tomato leaves, limited population growth of the bacterial pathogen *P. syringae*. This effect persisted across independent field sampling seasons and against two different strains of *P. syringae*.

Phage communities appeared to have a stronger effect in the experiments with the specialized tomato strain, PT-23, than in the experiments with the generalist strain DC3000. This might reflect differences between the two pathogen strains; for example, since DC3000 appears to grow more slowly in tomato leaves, it is possible that differences between treatments were just beginning to emerge when the plants were harvested at 24 h post-infection. Another possibility is that the plants grown in the field to generate microbial inocula contained fewer, or less active, lytic phages in the second year of field sampling. Of note, the size-selective filtration method used in this study is highly effective at excluding lytic phages, but may miss temperate phages located within host cells [35]. Environmental conditions such as heat and dry spells are common in the Sacramento Valley and can shift phage communities towards increased rates of lysogeny [59].

Phage communities could influence disease susceptibility through several possible mechanisms that we attempted to disentangle (Fig. 1). We first asked whether the phage communities we had sampled from the field contained, by chance, phages capable of directly infecting and killing *P. syringae*. Several phage communities contained phages that formed plaques on *P. syringae* plates. Some of them were particularly effective at limiting *P. syringae* colonization in the plant experiments as well, while others produced plaques in vitro but were not notably protective *in planta*. However, we continued to observe protective effects of the phage treatment when we excluded all phage communities with plaque-forming activity from analysis. We explored the possibility that the phage concentration process could accumulate bacterial small molecules, such as LPS, that are recognized by the plant immune system. Applying the corresponding quantities of purified LPS to plant leaves did not recapitulate the effect of the phage communities (Fig. S3).

In a final effort to control for any direct effects of phages or molecules present in the phage fraction on the plant host, we conducted an experiment that paired microbial communities with either their own phages or phages from increasingly distant sources—both of which would also capture any other components of concentrated phage fractions. This experiment had the additional aim of exploring how specific the effects of phage communities are across geographical and ecological distances. Phage communities consistently had a stronger effect on pathogen protection when they came from a neighboring plant of the same species than when they came directly from the same plant (Fig. 4), while phage communities from more distant sources were not notably protective. Amplicon sequencing and droplet digital PCR measurements of the microbial communities suggested that bacterial lysis and community turnover occurred at higher rates when bacteria were paired with their own phages than with neighboring phages. This is consistent with past reports that phages in nature are typically “ahead” of bacteria in coevolution; that is, sympatric phages are typically more infective than allopatric phages [60–62]. A possible explanation for our results is that moderate amounts of phage activity restrict dominant bacterial populations enough to allow other, potentially pathogen-protective bacteria to grow—i.e. frequency-dependent selection or the kill-the-winner hypothesis [32–34]—but high levels of phage lysis reduce bacterial populations to the point of actually leaving them more vulnerable to pathogen infection. Additional work will be needed to explicitly test for dose-dependent effects of phage communities, and to explore whether the impacts we observed on *P. syringae* population growth extend to disease progression.

Overall, our data point to an indirect yet important role for phage communities in plant defense against the pathogen *P. syringae*. This phenomenon was not attributable to direct phage-plant interactions or phage infection of *P. syringae*, as it was largely dependent on the presence and identity of the commensal microbiome. The presence of phages was most impactful when they originated from a nearby plant, rather than from the same plant as the microbial community – perhaps consistent with the ecological theory that intermediate levels of disturbance and predation produce the most diverse and resilient communities [63–65]. Together, this work highlights a need to consider phages in studies of microbiome-mediated resistance to bacterial pathogens, as they likely have untapped potential for our understanding of dysbiosis and disease.

## Supplementary Material

Debrayetal_SIFigures_ycaf065

Debrayetal_SITables_ycaf065

## Data Availability

The 16S rRNA sequences generated in this study are available on the NCBI Sequence Read Archive under the accession PRJNA1121198. Additional experimental data are located at the GitHub repository reenadebray/phage_depletion_microbiome.
