# Supplementary material for: Phages indirectly maintain tomato plant pathogen defense through regulation of the commensal microbiome
Source: ISME Commun. 2025 Apr 18;5(1):ycaf065. doi: 10.1093/ismeco/ycaf065 (PMC12066413; doi:10.1093/ismeco/ycaf065)
Supplement: Debrayetal_SIFigures_ycaf065 [file debrayetal_sifigures_ycaf065.docx]

Supplementary Information for **‘Phages indirectly maintain tomato plant pathogen defense through regulation of the commensal microbiome’**


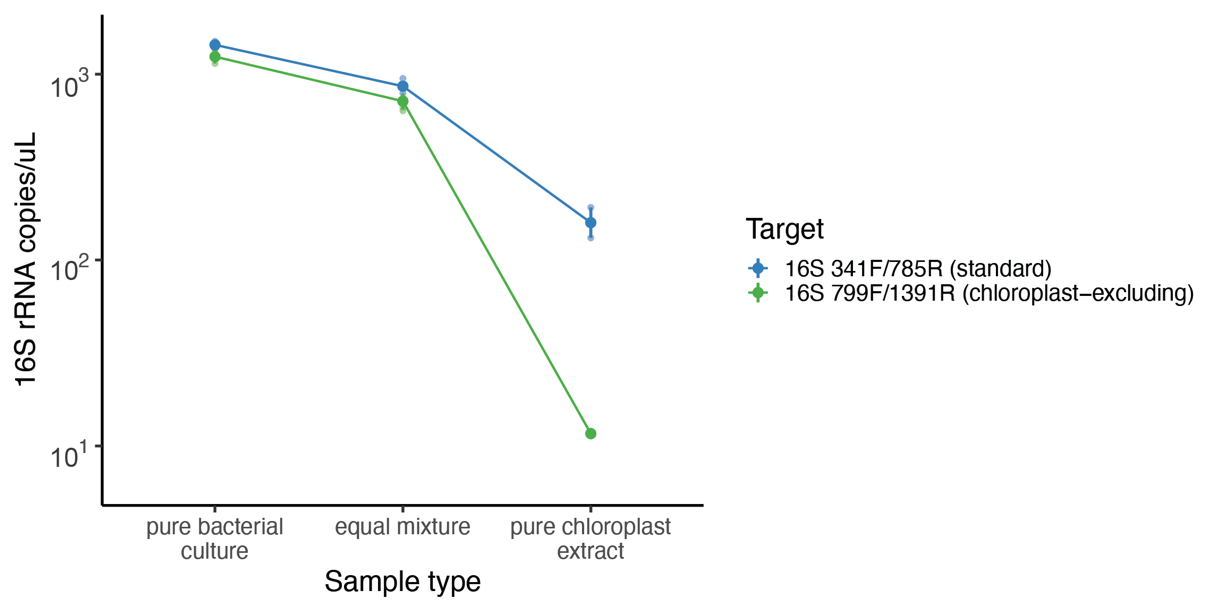


**Figure S1. Validation of chloroplast-excluding primers for bacterial abundance assay.** Droplet digital PCR was conducted on either an overnight bacterial culture of *Pseudomonas putida*, chloroplasts isolated from homogenized tomato leaves by differential centrifugation, or an equal mixture by volume of bacteria and chloroplasts.


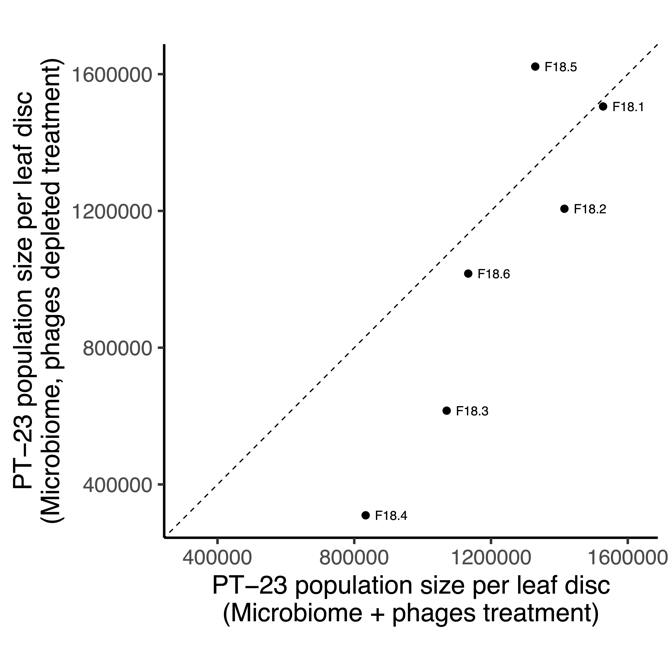


**Figure S2. Variation in *Pseudomonas syringae* colonization among biological replicates.** Population sizes of *Pseudomonas syringae* pv. tomato strain PT-23 on plants treated with microbial and phage communities (x-axis) or phage-depleted microbiomes (y-axis).


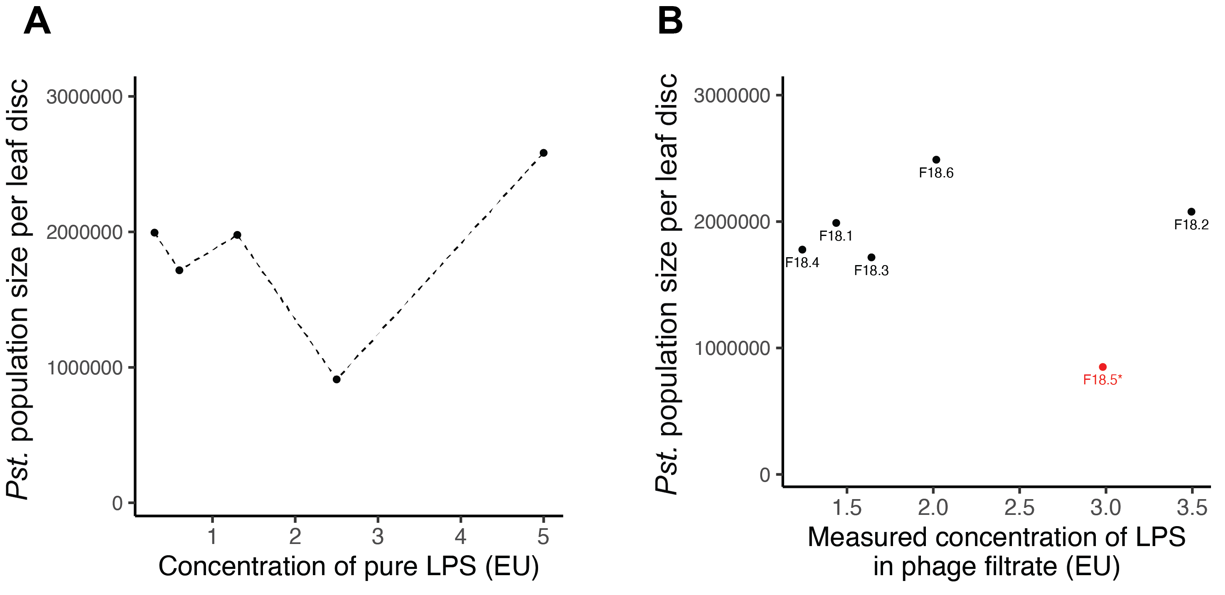


**Figure S3. Relationship between lipopolysaccharide content of treatment and *Pseudomonas syringae* colonization. (a)** Population sizes of *Pseudomonas syringae* pv. tomato strain PT-23 on plants treated with pure bacterial lipopolysaccharide (LPS). **(b)** LPS concentrations of phage filtrates isolated from an agricultural field plot in 2018, and resulting population sizes of *Pseudomonas syringae* pv. tomato strain PT-23 on plants treated with those phage communities. Red text with an asterisk indicates plaque-forming activity on agar plates of the respective strain of *Pseudomonas syringae.*

**
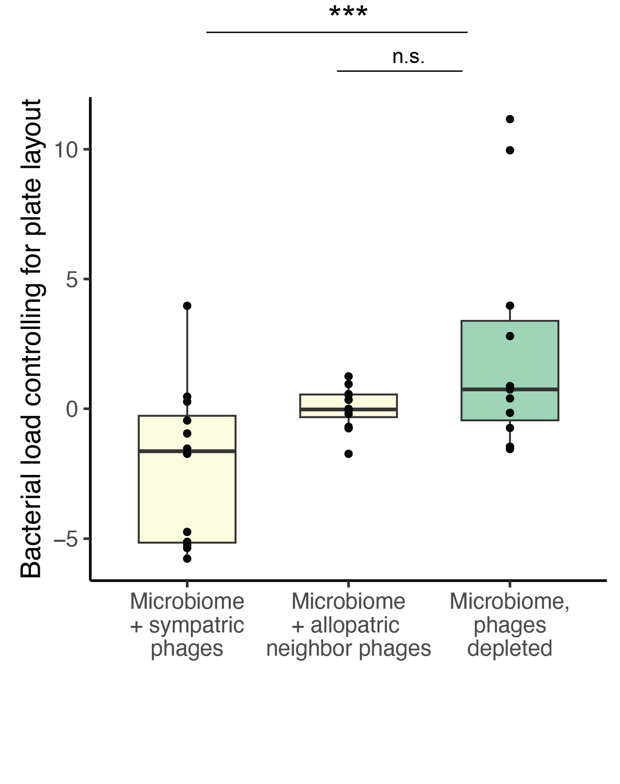
**

**Figure S4. Total abundance of microbial communities on leaves after microbiome and phage transplant.** Due to spatial effects in this droplet digital PCR assay, values represent 16S copy numbers after controlling for location on the ddPCR plate. Box-plots lines indicate the lower quartile, median, and upper quartile, and whiskers indicate 1.5 times the interquartile range.
